# Revision of the Protocol of the Telephone Triage System in Tokyo, Japan

**DOI:** 10.1155/2021/8832192

**Published:** 2021-04-21

**Authors:** Atsushi Sakurai, Jun Oda, Takashi Muguruma, Shiei Kim, Sachiko Ohta, Takeru Abe, Naoto Morimura

**Affiliations:** ^1^Division of Emergency and Critical Care Medicine, Department of Acute Medicine, Nihon University School of Medicine, 30-1 Oyaguchikamichou, Tokyo, Japan; ^2^Department of Emergency and Critical Care Medicine, Tokyo Medical University, Shinjuku City, Tokyo 160-8402, Japan; ^3^Department of Emergency Medicine, Yokohama City University Graduate School of Medicine, Kanazawa Ward, Yokohama City, Kanagawa 236-0027, Japan; ^4^Department of Emergency and Critical Care Medicine, Nippon Medical School, 1-1-5, Sendagi, Tokyo, Japan; ^5^Department of Pharmaceutical and Medical Business Sciences, Nihon Pharmaceutical University, 3-15-9 Yushima, Bunkyo City, Tokyo, Japan; ^6^Advanced Critical Care and Emergency Center, Yokohama City University Graduate School of Medicine, Kanazawa Ward, Yokohama City, Kanagawa 236-0004, Japan; ^7^Department of Acute Medicine, Graduate School of Medicine, The University of Tokyo, 7-3-1 Hongo, Bunkyo City, Tokyo, Japan

## Abstract

**Introduction:**

The Emergency Telephone Consultation Center in Tokyo (#7119) was the first telephone triage system in Japan and has operated since 2007. This study examined the revision of the #7119 protocol by referring the linked data to each code of the triage protocol.

**Methods:**

We selected candidates based on the medical codes targeted by the revision, linking data from the nurses' decisions in triage and the patients' condition severity when the ambulance arrived at the hospital, gathering data from June 1, 2016, to December 31, 2017. Then, several emergency physicians evaluated the cases and decided whether the code should be moved to the more or less urgent category or if new protocols and codes would be established.

**Results:**

In this revision, 371 codes were moved to the less urgent category, 35 codes were moved to the more urgent category, and 128 codes were newly established. In all, 59 red codes (transfer to the ambulance dispatcher) were reduced, while 254 orange codes (attendance at hospital within 1 hour) and yellow codes (within 6 hours) were moved to less urgent, and 12 yellow and green codes (within 24 hours) were moved to more urgent.

**Conclusion:**

We adjusted the triage codes for the revision by linking the call data with the case data. This revision should decrease the inappropriate use of ambulances and reduce the primary care workload. To achieve a more accurate revision, we need to refine the process of evaluating the validity of patients' acuity over the telephone during triage.

## 1. Introduction

In Japan, the number of patients transported to the hospital by ambulance is rising every year. Ambulance transport increased from 4,902,753 patients in 2007 to 5,736,086 in 2017 [1]—an increase of 833,333 patients and 17% over ten years. The majority of these patients are transported to hospitals by public ambulance cars. Therefore, this increase in emergency dispatch results in a financial burden on the public health system. Moreover, unnecessary ambulance transfers tie up resources, delaying their availability for life-threating cases; the challenge lies in adjusting the guidelines to regulate appropriate usage of a finite prehospital emergency system for patients. In many developed countries, population aging is increasing, which in turn contributes to the overload on the prehospital ambulance system. We seek a solution to this problem.

Inappropriate use of unnecessary emergency ambulance transfers is common [2]. To solve this problem, many countries, including Sweden [3], Australia [4], the United Kingdom [5], and the United States [6], have established telephone-based medical consultation services. These systems triage patients, assessing their conditions' urgency to allocate clinically appropriate prehospital medical resources. From this point of view, we established the new original algorithm and protocol of telephone triage at the #7119 center [7, 8], referring to the Manchester Triage System (MTS) [9] and telephone triage protocol for nurses at Portland, Oregon [10]. Finally, on June 1, 2007, the Tokyo Metropolitan Government began operating its Emergency Telephone Consultation Center (#7119 center), which is a 24/7 nurse-run telephone medical advice line that either refers callers to the most appropriate services or provides them with advice on how to care for their condition [7]. Before the establishment of the #7119 center, an ambulance was definitely dispatched if an emergency call was received as there was no call triage system at the dispatcher in Tokyo's ambulance system. The #7119 center was the first telephone triage system in Japan, serving a population of about 13 million citizens and operating computer-programmed medical protocols by the Emergency Telephone Consultation Center of the Committee of Emergency Medicine in the Tokyo Medical Association.

Several validated emergency scales dedicated to triage patients at emergency department (ED) admission points exist, such as the Canadian emergency department triage and acuity scale (CTAS), emergency severity index (ESI) [11], and MTS [9]. There have been many validation studies in the ED [12–15]. However, no validation study of the telephone triage protocol itself has been reported to date. Fortunately, we could access the entire database of telephone triage cases in Tokyo, and also of ambulance cases in Tokyo, as almost all ambulance dispatches transferring patients to hospitals are public. This is a descriptive study regarding the present situation of the #7119 telephone triage. The study also describes the revision of the code categories through formatted rules by linking the call data to the case data for each code.

## 2. Methods

This study was approved by the #7119 handling committee of the Tokyo Medical Association. We retrospectively reviewed the data of the Tokyo Fire Department (FD) from June 1, 2016, to December 31, 2017.

### 2.1. System of #7119

Several emergency doctors in Tokyo established the original protocol for the #7119 system. Under the #7119 system, each consultation is classified into one of five triage categories (red, orange, yellow, green, or blue) based on the perceived severity of a caller's condition [7, 8]. The triage process of #7119 consists of three steps. In Step 1, a call handler (nurse) receives a patient's call and takes information about the patients' identification and reason for calling. If certain keywords occur, especially cardiac arrest, no respiration, no pulse, submersion, or cold body, the call handler immediately connects the call to the emergency center to dispatch an ambulance (category red). In Step 2, the telephone consultation nurse asks the patient questions regarding the presence or absence of severe, abnormal physiological signs; this is much like a primary trauma survey [16]. In Step 3, there are 98 symptom-specific protocols for injuries or diseases, including 18 for pediatric cases. For each protocol, the consultation nurse asks a series of questions to determine the appropriate five-level triage category from red to green. Each question is designed to identify a specific condition that has a code. The triage category assigned may be based on the specific conditions revealed by these questions. In the management of #7119, a physician is always on call; the nurse can consult with the physician and, with agreement of physicians, change the initial case category to rank it up (more urgent than originally determined) or rank it down (less urgent). This is done when nurses doubt the appropriateness of the triage category indicated by the #7119 protocol. Rank-up cases formed the red category, wherein patients were taken to the hospital in an ambulance, while rank-down cases were the less urgent category, wherein the patients went to the hospital by themselves.

### 2.2. Severity of Patients at Hospitals

In this study, the severity of a patient's condition on emergency admission at a hospital is classified into one of five categories—dead, lethal, severe, moderate, and mild—by a physician and recorded publicly by the ambulance staff. This administrative guideline enabled us to acquire data on patients' condition severity for all patients transferred to the hospital by ambulance. Some of these cases were not assigned to the red category by the #7119 protocol but were ranked up to the red category by nurses (with the agreement of a physician) and then transferred to the hospital by ambulance. We were able to obtain patients' severity data from those codes of orange, yellow, and green.

### 2.3. Data Linked to Each Code

The total number of codes used in Step 3 was 1,327, of which 527 were red, 346 were orange, 255 were yellow, and 199 were green. Each code was linked with data on the total number of cases, rank-downs by nurses, transfers to the dispatcher, and patients' condition (dead, lethal, severe, moderate, or mild). Each code also linked comments from relevant consultation nurses and consulting physicians. These comments were reviewed by the protocol improvement working group for the #7119 handling committee of the Tokyo Medical Association.

### 2.4. Revision Rules for the Category of Each Code

In this revision of the system, we changed certain categories of codes, moved them to a more or less urgent category, and established new codes for use in Step 3 of the #7119 triage process. We established the following rules to select codes that we thought should be changed to other categories:

1. When the ratio of rank-down cases was over 30% (should be overtriaged)
2. When the percentage of mild cases receiving a red code ratio was over 70%, as calculated by the number of mild cases upon hospital arrival divided by the total number of cases transferred to the dispatcher (should be overtriaged)
3. When orange, yellow, or green codes were assigned to a case, the patient's condition on arrival was dead, lethal, or severe (should be undertriaged)
4. When emergency physicians felt the need to change the code category based on their clinical evaluations

For code selections described by 1, 2, or 4, we checked the data on severity and excluded codes as candidates to be moved to the less urgent category if there were cases with the conditions of dead, lethal, or severe in the linked data. On the contrary, code selections described by 3 required the candidate to move to the red category. The final decision to revise a code's category to more or less urgent was made after a review by several emergency physicians of protocol improvement working group members on the #7119 handling committee of the Tokyo Medical Association. New symptoms or codes were established based on this conference.

## 3. Results

### 3.1. The Present Situation of the #7119 Telephone Triage

As shown in Table 1, in this study period, in Step 3, there were 196,119 total calls that were linked to codes. Of these, 71,389 patients were allocated to the red category, 61,179 to the orange category, 41,050 to the yellow category, and 22,501 to the green category. Among the red category calls, 19,939 (27.9%) were ranked down by nurses, and 29,039 (40.7%) were transferred to dispatchers to send an ambulance. Among those (red code) cases that were transferred to dispatchers, 18,662 were diagnosed as mild and 8,710 as moderate when patients arrived at the hospital. Finally, 485 cases were classified as severe (412), lethal (65), and dead [8]. In the orange category, 1,347 cases were reallocated to red with the agreement of a consulting physician; 157 yellow and 43 green allocations were also reallocated to red. In these cases, 8 cases were classified as severe upon hospital arrival. Four codes linked 8 of these cases and were picked up to candidates after changing the category to red code.

### 3.2. The Revision of the Code Categories

As seen in Table 2, we moved 371 codes to the less urgent category and 35 to the more urgent category. In total, 117 codes were moved from red to other categories, and 23 codes were moved to the upper category and from other categories to red. A total of 17,752 cases were linked by codes from red to other codes (red to orange: 16,368; red to yellow: 1,373; red to green: 11), and there were 41,273 other cases (orange to yellow: 30,635; orange to green: 1,418; yellow to green: 9,220). On the contrary, there were 3,518 cases that were moved from less urgent categories to red (orange to red: 3478 and yellow to red: 40) and 852 cases that were moved from less urgent categories to more urgent categories other than red (yellow to orange: 795 and green to yellow: 57).

One new symptom, “nonmovable,” was added to the new codes. This protocol was defined for cases in which the patients could not be moved without causing cardiac arrest, trauma, disturbance of consciousness, palsy, or pain. This symptom was supposed to accord with the patients' state of preshock or hypokalemia. We added two codes in the protocol for ‘numbness,' with the symptom of numbness being assumed to indicate a stroke attack. The Cincinnati Prehospital Stroke Scale (CPSS) is used to detect potential strokes by evaluating facial droop, arm drift, and speech [17]. However, the original protocol included only a code with speech, so we added two new codes related to facial droop and arm drift. Through similar refinements, we established a total of 128 new codes.

Information regarding the types of codes moved can be accessed from the supplementary data (available here).

## 4. Discussion

Out of the total 196,119 calls to #7119 during the study period, 30,556 calls were transferred to the dispatcher to use an ambulance. A total of 165,563 (84.3%) calls were advised against the use of an ambulance. If there is no telephone triage system like #7119, ambulances would have been dispatched for all these cases. Considering the nurses' telephone triage, the estimated decrease in costs associated with dispatching an ambulance was ¥7,450,335,000 ($67,730,318; $1 = ¥110). This was calculated based on the decrease in the number of dispatched ambulances (165,563) and the expenditure to dispatch one ambulance (¥45,000) [7]. Fortunately, we can access the entire data of ambulance cases in Tokyo as almost all ambulance dispatches transferring patients to hospitals were public, and we could estimate the financial effect of the #7119 center. However, among the red code cases, only 485 cases were diagnosed as severe, lethal, or dead. Therefore, we should revise the protocol of the #7119 center.

A medical telephone triage system does not always reduce the primary care workload. Campbell et al. reported, based on their pragmatic, cluster-randomized trial of a large population in England, that telephone triage by general physicians and nurses increased primary care contacts compared with usual care [18]. They concluded that the telephone triage was associated with a redistribution of the primary care workload. Their study showed that while it may be difficult to reduce the care workload through telephone triage, it may be possible to adjust it for improved efficiency. In this revision, we moved 117 codes from red to other categories, moved 23 codes to red, and established 35 new red codes. The total number of red codes decreased from 59 (10.5%) from the previous protocol to 527 in the new protocol. The revised red-code protocol may decrease the number of clinically inappropriate ambulance transfers. Furthermore, 254 orange and yellow codes were moved to the less urgent category; 12 yellow and green codes were moved to more urgent categories, and 35 new orange codes and 53 new yellow codes were added. These revisions should reduce the primary care workload.

With telephone triage, undertriage is a critical issue that can compromise patient safety. One study showed that, on average, about 10% of telephone triage contacts resulted in unsafe care advice, including undertriage, in real patients [19]. However, Gamst-Jensen et al. [20] reported that 0.04% of all calls to an out-of-hours telephone service in Denmark involved undertriage, a much lower incidence. In our revision, we identified eight cases of undertriage and adjusted the associated linked codes in #7119 to red codes. These cases were extracted from the orange, yellow, and green categories through the up-code process, and we were able to see the results from ambulance records; this led us to adjust the #7119 protocol. We may need a warning system to highlight undertriage cases in the telephone triage system. This would be an automatic process that would flag cases with unfavorable code-outcome mismatches.

We identified codes associated with overtriage, selecting those for which over 30% of the cases were ranked down by nurses, which they can do with a physician's agreement. Rank-down cases can be indications of overtriage by nurses and physicians. In the data used in the present study, 27.9% of all the red category cases were ranked down, allowing us to evaluate codes for their possibility of leading to overtriage. Another way we identified which red codes led to overtriage was the ambulance records noting patients' condition severity. We found that 64% of the red-coded patients (compelling ambulance transfer) turned out to be mild upon hospital arrival, and we selected codes where this rate was over 70% as codes that could be down-ranked. In this manner, we evaluated the codes for the validity of triage.

Lake et al. reported evidence of telephone triage across nine key indicators: access, appropriateness, compliance, patient satisfaction, cost, safety, health service utilization, physician workload, and clinical outcomes [21]. In our study, we evaluated over- or undertriage by the clinical outcome of patients on ambulance hospital arrival. However, the best way to evaluate the validity of patients' acuity through telephone triage is still debated. One study focused on “paramedic treatments” such as drug or fluid administration, airway management, perfusion or cardiovascular support management, and mental health management as indications of ambulance usage [22]. Further discussion and investigation are needed in other methods for evaluating the urgency of callers' conditions to ensure accurate triage results by nurses.

Our study has a limitation. The revision of codes should decrease the number of cases where an ambulance is used but not warranted, and this may reduce the primary care workload from what it was before the revision for those patients using #7119. However, this could not be proven. To confirm this, we will need to investigate the real population of patients in each category and compare the workloads of a medical institute in the local area before and after the protocol revision.

## 5. Conclusion

We revised the #7119 protocol using the data linked by the triage code and adjusted codes associated with over- or undertriage to more optimal categories. The revised protocol should decrease the number of cases of clinically inappropriate ambulance transfers and promote the reduction of the primary care workload by nurse's telephone triage.

## Figures and Tables

**Table 1:** Basic data on case numbers linked with the code for each triage category in #7119.

Category (amount of codes)	Red (527)	Orange (346)	Yellow (255)	Green (199)	Total (1,327)
Total number of patients	71,389	61,179	41,050	22,501	196,119
Ranked down by nurses (%)	19,939 (27.9)	11,340 (18.5)	3,186 (7.8)	2,006 (8.9)	36,471 (18.6)
Transfer to dispatchers	29,039 (40.7)	1,347 (2.2)	157 (0.4)	43 (0.2)	30,556 (15.6)

*Outcome at arrival to the hospital*					
Dead	8	0	0	0	8
Lethal	65	0	0	0	65
Severe	412	7	1	0	420
Moderate	8,710	332	30	10	9,082
Mild	18,662	943	115	28	19,748
Patient refusal of transfer to the hospital	1,182	65	11	5	1,263

**Table 2:** Number of revised codes with category and of patients linked by each code.

	Number of codes	Number of patients linked with these codes
*Code moved to the less urgent category*		
Red to orange	96	16,368
Red to yellow	20	1,373
Red to green	1	11
Orange to yellow	175	30,635
Orange to green	16	1,418
Yellow to green	63	9,220
Sum of rank-downs	371	59,024

*Code moved to the more urgent category*		
Orange to red	22	3,478
Yellow to red	1	40
Green to red	0	0
Yellow to orange	8	795
Green to orange	0	0
Green to yellow	4	57
Sum of rank-ups	35	4370

*New code*		
Red	35	
Orange	35	
Yellow	53	
Green	5	
Sum of new codes	128	

## Data Availability

The excel data used to support the findings of this study may be released upon application to the #7119 handling committee of the Tokyo Medical Association who can be contacted at sakurai.atsushi@nihon-u.ac.jp.

## Supplementary Materials

Supplementary Materials We show three kinds of supplementary files about codes moved to less urgent, codes moved to more urgent, and new codes.
