# Supplementary material for: Revision of the Protocol of the Telephone Triage System in Tokyo, Japan
Source: Emerg Med Int. 2021 Apr 21;2021:8832192. doi: 10.1155/2021/8832192 (PMC8081606; doi:10.1155/2021/8832192)
Supplement: Supplementary Materials — We show three kinds of supplementary files about codes moved to less urgent, codes moved to more urgent, and new codes. [file 8832192.f1.zip › 8832192.f1/Supplement 1 Code moved to less urgent.docx]

| No | ProtcolFileNo | Protocol name | Code | Change category from A to B |
| --- | --- | --- | --- | --- |
| 1 | 4 | Palpitations | I have a less amount of urine. OR My urine is getting darker. The patient has a less amount of urine. OR His/Her urine is getting darker. Less amount of urine. OR Darker urine. | From Red to Orange |
| 2 | 4 |  | I have dry skin/lips. The patient has dry skin/lips. Dry skin/lips. |  |
| 3 | 4 |  | I have terrible thirst. The patient has terrible thirst. Terrible thirst. |  |
| 4 | 7 | Headache | I have blurry vision. OR I have other vision problems (black spots, unusual view, etc.). |  |
| 5 | 8 | Chest pain | Did you recently injured yourself or recently get involved in a traffic accident? |  |
| 6 | 8 |  | I take the oral contraceptive. The patient takes the oral contraceptive.  Oral contraceptive taken. |  |
| 7 | 8 |  | I have a pain, swelling, bulges, a reddish tinge or feverishness in the foot/feet. OR I have pedialgia.  The patient has a pain, swelling, bulges, a reddish tinge or feverishness in the foot/feet.  Foot/Feet trouble (i.e., pain, swelling, bulges, reddish tinge or feverishness). |  |
| 8 | 9 | Back pain | I see blood in my urine, and I have a severe pain when urinating.   My urine is red. I have a strong pain toward the urination.  There is blood mixed in the urine. OR The patient has a severe pain when urinating.  Blood in the urine. OR Severe pain in urination. |  |
| 9 | 11 | Low back pain | I cannot move because of a bad pain. OR I cannot visit a hospital by myself.  The patient cannot move in a bad pain. OR The patient cannot go to the hospital by himself/herself.  Unable to move in a bad pain. OR Unable to go to the hospital by oneself. |  |
| 10 | 11 |  | I have blood in my urine. OR I have a bad pain with urination. There is blood mixed in the urine. OR The patient has a sharp pain while passing water. Blood mixed in the urine. OR Sharp pain in passing water. |  |
| 11 | 12 | Syncope | The patient has injury. OR The patient injured by syncope. |  |
| 12 | 12 |  | The patient has cardiac disease (AMI, angina, arrythmia or cardio myopathy). OR The patient is under treatment with DM. |  |
| 13 | 14 | Pyrexia | I have dry skin/lips. The patient has dry skin/lips. Dry skin/lips. |  |
| 14 | 14 |  | I have a less amount of urine. OR My urine is getting darker. The patient has a less amount of urine. OR His/Her urine is getting darker. Less amount of urine. OR Darker urine. |  |
| 15 | 14 |  | I felt dizzy on standing up. The patient felt dizzy on standing up. Dizziness on standing up. |  |
| 16 | 15 | Rash | Do you have spots of bleeding/bruising or purple spots on your skin? |  |
| 17 | 18 | Constipation | Do you see a lump or protrusion at your groin or abdomen? |  |
| 18 | 19 | Diarrhea | I have a less amount of urine. OR My urine is getting darker. The patient has a less amount of urine. OR His/Her urine is getting darker. Less amount of urine. OR Darker urine. |  |
| 19 | 19 |  | I felt dizzy on standing up. The patient felt dizzy on standing up. Dizziness on standing up. |  |
| 20 | 20 | Vomiting | I have a bloated stomach. The patient has a bloated stomach. Bloated stomach. |  |
| 21 | 20 |  | I recently got injured my head/chest/abdomen, or got a high energy impact on my head/chest/abdomen.  The patient recently got injured in the head/chest/abdomen, or physically received a forceful strike.  Recent injury in the head/chest/abdomen. OR Forceful strike physically given. |  |
| 22 | 24 | Difficulty Urination | Severe pain with urination. |  |
| 23 | 25 | Urine color Abnormal | Red and large amount of urine |  |
| 24 | 28 | Genital Urinary problem | Penis or scrotum is sweling and with pain |  |
| 25 | 28 |  | The patient can't stop penis bleeding |  |
| 26 | 28 |  | Stucking foreign body in urethra |  |
| 27 | 30 | Hearing difficulty | Vertigo (diziness) or nausea |  |
| 28 | 32 | Dizziness, Vertigo | I have a bad dizziness. The patient has a bad dizziness. Bad dizziness. |  |
| 29 | 32 |  | I have diarrhea/vomiting. The patient has diarrhea/vomiting. Diarrhea. OR Vomiting. |  |
| 30 | 32 |  | I have nausea. The patient has nausea.  Nausea. |  |
| 31 | 32 |  | I am under treatment of high blood pressure. OR I have a diagnosis of high blood pressure.   The patient has been treated for high blood pressure. OR The patient suffered from high blood pressure.   Under treatment for high blood pressure. OR High blood pressure in the past. |  |
| 32 | 33 | Numbness | My hands and feet are colder/paler than other parts.  The patient's hands and feet are colder/paler than other parts.  Colder/Paler hands and feet. |  |
| 33 | 35 | Breast pain | History of cardiac disease, especially AMI and angina. |  |
| 34 | 38 | Hypertension | Confusion, dizziness or lightheadedness. |  |
| 35 | 38 |  | Severe generalized weakness. |  |
| 36 | 41 | Anxiety | Violence or intention of violence to to others |  |
| 37 | 41 |  | Suicidal ideation and acknowledgment of a specific sicidal plan |  |
| 38 | 45 | Nose injury Nose bleed | Headache or eye pain after nose injury |  |
| 39 | 46 | Mouth and Tooth problem | Severe pain on tongue, lips, patella, or gums (regardless of traumatic episode) |  |
| 40 | 46 |  | Oral pain AND facial swelling |  |
| 41 | 46 |  | Tooth injury or tooch extraction AND bleeding with despite compression for 10 minutes |  |
| 42 | 46 |  | Toothache AND heart disease |  |
| 43 | 47 | Hand/Wrist problem | Severe pain |  |
| 44 | 47 |  | Deformity or abnormal direction of a finger |  |
| 45 | 47 |  | Ongoing bleeding |  |
| 46 | 48 | Leg problem | Numb of leg suddenly |  |
| 47 | 48 |  | Ongoing bleeding |  |
| 48 | 51 | Laceration | Visible bone in laceration |  |
| 49 | 55 | Bites | Palpitation |  |
| 50 | 55 |  | Site of animal (NOT insect) bite at head, face or neck |  |
| 51 | 59 | Head injury | Ongoing bleeding at head or nose |  |
| 52 | 60 | Eye injury | Injury on eye(s) or eyelid(s) |  |
| 53 | 60 |  | Pain or blurry vision after exposure to chemicals |  |
| 54 | 62 | Neck and Back injury | Ongoing bleeding from injury site |  |
| 55 | 63 | Trunk injury | Ongoing bleeding from injury site |  |
| 56 | 64 | Hand, wrist, foot, leg and face injyry | Severe pain |  |
| 57 | 64 |  | Ongoing bleeding from injury site |  |
| 58 | 65 | Foreign body, swallowing of solid mass | Impossibility taking out of an object smaller than 1-yen coin from throat |  |
| 59 | 65 |  | The patient may have ingested some toxin. |  |
| 60 | 65 |  | Vomiting. OR Nausea. |  |
| 61 | 66 | Foreign body, swallowing of liquid | continuity of coughing. |  |
| 62 | 68 | Overdose | Diarrhea |  |
| 63 | 69 | Foreign body Eye | Pain or blurry vision by a some drug into eye |  |
| 64 | 73 | Foreign body, Rectum | Severe pain |  |
| 65 | 76 | Food poisoning | I have a less amount of urine. OR My urine is getting darker. The patient has a less amount of urine. OR His/Her urine is getting darker. Less amount of urine. OR Darker urine. |  |
| 66 | 76 |  | Dry skin/lips |  |
| 67 | 76 |  | Feeling thirst |  |
| 68 | 76 |  | I felt dizzy on standing up. The patient felt dizzy on standing up. Dizziness on standing up. |  |
| 69 | 76 |  | Cring without tear at a pediatric patient |  |
| 70 | 77 | Heat stroke | Feverish AND headache |  |
| 71 | 77 |  | Feverish AND nausea |  |
| 72 | 77 |  | Feverish AND rapid pulsation or rapid respiration |  |
| 73 | 77 |  | High fever (>39℃ ) |  |
| 74 | P2 | Convulsion Child | First episode of seizure in his/her life. |  |
| 75 | P2 |  | Repeated seizure |  |
| 76 | P2 |  | Asymmetric movement during the seizure. |  |
| 77 | P2 |  | Agitated OR raging |  |
| 78 | P3 | Cough Child | Chest pain |  |
| 79 | P5 | Asthma Child | No improvement of symptom even with his/her prescribed medications (inhaler, oral medications) |  |
| 80 | P5 |  | Difficulty swallowing water |  |
| 81 | P6 | Shortness of breath Child | Chest pain |  |
| 82 | P8 | Vomiting Child | Continuous vomiting (6 times or over, vomiting several times even after no solid things (food) can be found in the vomit) |  |
| 83 | P8 |  | Vomitting with blood or greenish liquid. |  |
| 84 | P8 |  | Body temperature of 38℃ or higher |  |
| 85 | P8 |  | (Baby) Throwing up several times after drinking milk |  |
| 86 | P8 |  | Cring without tear |  |
| 87 | P8 |  | Strong bang on the abdomen |  |
| 88 | P9 | Diarrhea Child | Exhausted and weak |  |
| 89 | P9 |  | Severe vomiting or severe abdominal pain |  |
| 90 | P10 | Abdominal pain Child | The patient cannot walk because of severe abdominal pain |  |
| 91 | P10 |  | Blood component in the vomitus (red:fresh bleeding, black: somewhat old bleeding) |  |
| 92 | P11 | Constipation Child | Continuous vomiting (6 times or over, vomiting several times even after no solid things (food) can be found in the vomit) |  |
| 93 | P15 | Tobacco ingestion Child | Eating wihtin 4 hrs AND ≧ 2cm |  |
| 94 | P15 |  | Agitated |  |
| 95 | P16 | Crying Child | The patient looks pale and cries intermittently due to abdominal pain (Cries severely, then stops crying as if patient were falling asleep. Patient again cries periodically). |  |
| 96 | P18 | Head injury Child | Vertigo or dizziness |  |
| 97 | 11 | Low back pain | The patient start pain after fall on his/her low back/buttock. | From Red to Yellow |
| 98 | 11 |  | Red (or bloody) urine after hitting low back/buttock |  |
| 99 | 14 | Pyrexia | Terrible thirst |  |
| 100 | 14 |  | Pain in the back or the side. |  |
| 101 | 19 | Diarrhea | Dry skin/lips |  |
| 102 | 19 |  | Terrible thirst |  |
| 103 | 23 | Urination pain | Severe pain with urination |  |
| 104 | 28 | Genital Urinary problem | Persistent erection of the penis for more than 30 minutes |  |
| 105 | 29 | Ear pain Ear discharge | Evident swelling around ear |  |
| 106 | 29 |  | Severe pain, even taking analgesics |  |
| 107 | 29 |  | Reddish swelling on the one side of face |  |
| 108 | 31 | Ear ringing | Vertigo or dizziness OR nausea |  |
| 109 | 38 | Hypertension | History of disease about abdominal or thoracic aorta |  |
| 110 | 38 |  | Bloody sputum |  |
| 111 | 39 | Hiccup | Continuous pain in abdomen or chest by hiccup |  |
| 112 | 46 | Mouth and Tooth problem | Tooth injury or tooch extraction AND treatment of anticoagulation drug |  |
| 113 | 51 | Laceration | Laceration for more than 10 cm |  |
| 114 | 78 | Hypothermia | Shivering, even with aggressive rewarming |  |
| 115 | P9 | Diarrhea Child | Cring without tear |  |
| 116 | P18 | Head injury Child | Tilting head psition OR head turning sideway |  |
| 117 | 46 | Mouth and Tooth problem | Tooth injury or tooch extraction AND treatment of anticoagulation drug |  |
| 118 | 1 | Shortness of breath | Injury or operation (Recently | From Orange to Yellow |
| 119 | 1 |  | Allergic history |  |
| 120 | 1 |  | Coughing OR yellowish or greenish phlegm |  |
| 121 | 2 | Wheezing | History of asthma |  |
| 122 | 2 |  | Coughing OR yellowish or greenish phlegm |  |
| 123 | 2 |  | Allergic history |  |
| 124 | 2 |  | History of chronic heart failure OR heart disease OR pulmonary embolism OR deep vein thrombosis OR kidney disease |  |
| 125 | 3 | Asthma | Throat pain, or common cold symptoms (ex., fever, chill, headache, cough, runny nose, etc.) |  |
| 126 | 3 |  | Traetment of steroid OR Inhaled drug |  |
| 127 | 3 |  | The symptom appeared after vomiting or chokin. |  |
| 128 | 3 |  | Hospitalization for asthma in the past (frequently or several times) |  |
| 129 | 3 |  | ICU treatment in the past |  |
| 130 | 4 | Palpitations | Headache |  |
| 131 | 4 |  | History of thyroid disease |  |
| 132 | 4 |  | History of heart disease |  |
| 133 | 4 |  | This symptom often appears. |  |
| 134 | 4 |  | Vomiting and/or diarrhea |  |
| 135 | 5 | Disturbance of consciousness | Alcoholic now OR alcoholic in the past |  |
| 136 | 5 |  | Dry skin or lips |  |
| 137 | 5 |  | Terrible thirst |  |
| 138 | 5 |  | Dizziness on standing up |  |
| 139 | 6 | Convulsion | The patient has run out of the drug for convulsions. |  |
| 140 | 7 | Headache | Body temperature of 38℃ or higher |  |
| 141 | 7 |  | Common cold symptoms (ex., fever, chill, headache, cough, runny nose, etc.) |  |
| 142 | 7 |  | The prescription drug does not work. |  |
| 143 | 7 |  | Hospital treatment for headache in the past OR History of migraine headache, tension headache or a cluster headache. |  |
| 144 | 9 | Back pain | Difficulty of urination OR unable to urinate |  |
| 145 | 9 |  | Frequent urination OR Pain with urination |  |
| 146 | 11 | Low back pain | Body temperature of 38℃ or higher AND nausea and/or vomiting |  |
| 147 | 11 |  | With trigger for my pain, such as lifting a heavy object |  |
| 148 | 11 |  | Difficulty of urination OR unable to urinate |  |
| 149 | 11 |  | Pain with urination |  |
| 150 | 12 | Syncope | After loss of consciousness AND clear consciousness now |  |
| 151 | 12 |  | Without loss of consciousness AND premonition of syncope or loss of consciousness |  |
| 152 | 13 | Common cold | With treatment of major disease (ex., heart disease, hepatic disease, DM, steroid treatment, cancer or other infectious disease, etc.), resulting in Immunodeficiency |  |
| 153 | 13 |  | Dry skin or lips |  |
| 154 | 13 |  | Terrible thirst |  |
| 155 | 14 | Pyrexia | Headache |  |
| 156 | 14 |  | Difficulty in swallowing food OR throat pain |  |
| 157 | 14 |  | Blackish/yellowish/greenish phlegm |  |
| 158 | 14 |  | Pyrexia without effectivity of antipyretics |  |
| 159 | 14 |  | Abdominal pain |  |
| 160 | 14 |  | Nausea |  |
| 161 | 15 | Rash | Body temperature of 38℃ or higher AND severe itch |  |
| 162 | 17 | Abdominal pain | Vomiting OR nausea |  |
| 163 | 17 |  | Body temperature of 38℃ or higher |  |
| 164 | 17 |  | Diarrhea |  |
| 165 | 17 |  | Dizziness on standing up |  |
| 166 | 17 |  | Palpable lump on Inguinal resion |  |
| 167 | 17 |  | Vaginal bleeding |  |
| 168 | 18 | Constipation | Vomiting OR severe abdominal distention |  |
| 169 | 18 |  | Tolerable, but spend quite hard time |  |
| 170 | 19 | Diarrhea | Abdominal pain |  |
| 171 | 19 |  | Vomiting OR nausea |  |
| 172 | 20 | Vomiting | Frequent diarrhea |  |
| 173 | 20 |  | Dry skin or lips |  |
| 174 | 20 |  | Terrible thirst |  |
| 175 | 20 |  | Body temperature of 38℃ or higher |  |
| 176 | 22 | Hematemesis Bloody stools | Hard to stop bleeding OR treatment of anticoagulation drug |  |
| 177 | 24 | Difficulty Urination | Dry skin or lips |  |
| 178 | 24 |  | Terrible thirst |  |
| 179 | 25 | Urine color Abnormal | Pain with urination |  |
| 180 | 25 |  | Difficulty of urination |  |
| 181 | 25 |  | Darker urine AND less amount of urine |  |
| 182 | 25 |  | Darker urine AND dry skin or lips |  |
| 183 | 25 |  | Darker urine AND terrible thirst |  |
| 184 | 27 | Vaginal bleeding | Severer pain than menstruation |  |
| 185 | 29 | Ear pain Ear discharge | Bloody component in a purulent discharge from an ear hole. |  |
| 186 | 29 |  | Deformity of ear |  |
| 187 | 29 |  | Tenderness around ear |  |
| 188 | 32 | Dizziness, Vertigo | Injury within 2 days |  |
| 189 | 32 |  | History of DM |  |
| 190 | 32 |  | Hard of hearing OR buzzing OR pain in the ear |  |
| 191 | 33 | Numbness | Hard of hearing OR buzzing in the ears |  |
| 192 | 33 |  | Intolerable numbness OR severe pain |  |
| 193 | 33 |  | Recently, lift with heavy item OR excessive exercise |  |
| 194 | 34 | Neck pain | Just after injury |  |
| 195 | 36 | Itch | History of same symptom |  |
| 196 | 36 |  | Pyrexia AND strong itch |  |
| 197 | 36 |  | Systemic rash OR rash on limbs |  |
| 198 | 36 |  | Pyrexia AND pain of throat or joint |  |
| 199 | 36 |  | Severe pain around rash |  |
| 200 | 38 | Hypertension | Dizziness after taking a new antihypertensive/blood pressure drug |  |
| 201 | 40 | Hyperventilation | Continuous high body temperature after antipyretic treatment (antipyretic drug, ice pillow etc.) at home |  |
| 202 | 46 | Mouth and Tooth problem | Injury at tongue, lip, palate (around the upper jaw), gums OR gingiva |  |
| 203 | 46 |  | Tooth injury or tooch extraction AND stop bleeding with compression |  |
| 204 | 47 | Hand/Wrist problem | The patient cannot remove a ring from the injured finger. |  |
| 205 | 48 | Leg problem | Swelling leg with fever OR warmth |  |
| 206 | 48 |  | Pain and coldness on thigh and/or lower leg at rest |  |
| 207 | 49 | Foot problem | Swelling ankle with fever OR warmth |  |
| 208 | 49 |  | Pain and coldness on foot at rest |  |
| 209 | 50 | Bleeding | Stab injury on limb AND bleeding was already stopped |  |
| 210 | 50 |  | History of diseases that make it difficult for blood to stop OR History of episode that make it difficult for blood to stop |  |
| 211 | 51 | Laceration | Bleeding diathesis OR under treatment of anticoagulation drug |  |
| 212 | 51 |  | Laceratiom trough eyelid, eyebrows or lip border |  |
| 213 | 51 |  | Unable to move of injured part by pain |  |
| 214 | 51 |  | Laceration through a joint |  |
| 215 | 51 |  | Foreign body in a wound |  |
| 216 | 52 | Bruise wound | Bleeding diathesis OR under treatment of anticoagulation drug |  |
| 217 | 52 |  | Severe swelling at bruise wound |  |
| 218 | 54 | Penetrating injury | Bleeding diathesis OR under treatment of anticoagulation drug |  |
| 219 | 54 |  | Palpitation |  |
| 220 | 56 | Burns | Burn wound larger than the patient's hand, except sunburn |  |
| 221 | 56 |  | Blisters on the patient's hand(s) or scrotum. |  |
| 222 | 56 |  | Severe swelling OR blister(s). larger than the patient's hand |  |
| 223 | 56 |  | Broken blister(s), larger than the size of patient's hand (including sunburn) |  |
| 224 | 60 | Eye injury | Continuous symptom such as swelling, pain, or uncontrollable tearing , > 30 minutes. |  |
| 225 | 60 |  | Injury caused by hot water or foreign body and pain persist after home care (eye wash) |  |
| 226 | 61 | Ear Injury Foreign body | Bleeding OR pain after removal of foreign body (including after grinding an earpick) |  |
| 227 | 65 | Foreign body, swallowing of solid mass | No information about foreign body |  |
| 228 | 67 | Aspiration, Gas Liquid | Able to speak OR to cough |  |
| 229 | 69 | Foreign body Eye | Still exist in eye |  |
| 230 | 69 |  | Pain, swelling, feverish OR watery eye |  |
| 231 | 70 | Contact lens problem | Unable to remove contact lense |  |
| 232 | 70 |  | Hard lens broken in eye |  |
| 233 | 70 |  | Pain after insertion of lenses that were not properply cleaned and rinsed before insertion |  |
| 234 | 70 |  | Persistant pain that is unresponsive to analgesic drugs and eye drops |  |
| 235 | 71 | Foreign body Nose cavity | Unable to remove a foreign body, trying several times by air push with exhalation, closing another nose hole |  |
| 236 | 71 |  | Stimulus OR sticky foreign body |  |
| 237 | 71 |  | Foreign body OR liquid OR blood to mouth |  |
| 238 | 71 |  | Infants (<6ys) |  |
| 239 | 72 | Throat Fish bone | Unable to remove a fish bone OR escalation of pain |  |
| 240 | 73 | Foreign body, Rectum | Something stinky OR some foreign body from ass hole OR buttock injury |  |
| 241 | 73 |  | Contineous bleeding from ass hole (not so much) |  |
| 242 | 74 | Foreign body Vagina | Possibility of pregnancy |  |
| 243 | 74 |  | Unable to remove foreign body, even with several trial |  |
| 244 | 74 |  | Swelling AND tenderness |  |
| 245 | 74 |  | Something stinky OR some foreign body from vagina |  |
| 246 | 74 |  | Using tampon for more than 1 day |  |
| 247 | 74 |  | Unable to remove contraceptive OR pkeasure device |  |
| 248 | 74 |  | Sudden increase of vaginal discharge |  |
| 249 | 75 | Foreign body Skin | By this injury foreign substances (piece of glass, plastic OR metal) is stinged OR remained in the skin |  |
| 250 | 75 |  | Fishhook OR nail is stinged |  |
| 251 | 75 |  | Foreign body deeply stinged in joint |  |
| 252 | 76 | Food poisoning | Continuous abdominal pain |  |
| 253 | 76 |  | Continuous nausea OR vomit |  |
| 254 | 76 |  | Continuous diarrhea |  |
| 255 | 76 |  | Blood in stool |  |
| 256 | 76 |  | Rash |  |
| 257 | 76 |  | Pyrexia |  |
| 258 | 77 | Heat stroke | Looks feverish AND act in a hot environment or excessive exercise |  |
| 259 | 78 | Hypothermia | Cool AND dry skin |  |
| 260 | P1 | Pyrexia Child | Insufficient fluid intake (decreaseing times OR amount of fluid intake) |  |
| 261 | P1 |  | Less amount of urine OR darker urine |  |
| 262 | P2 | Convulsion Child | Prophylactic administration of bennzodiazepine (such as DIAPP® suppository) AND no more stock of medications for it |  |
| 263 | P3 | Cough Child | Abnormal cough like dog OR seal barking |  |
| 264 | P3 |  | Consecutive coughs, followed by a whistle-like sound with inhaling |  |
| 265 | P3 |  | Less amount of urine OR darker urine |  |
| 266 | P3 |  | Consecutive coughs AND vomiting |  |
| 267 | P5 | Asthma Child | Less amount of urine or darker urine OR severe thirst |  |
| 268 | P5 |  | History of acute deterioration of asthma |  |
| 269 | P7 | Rash Child | Systemic wheal formation AND itchy |  |
| 270 | P7 |  | Unable to sleep by itchy |  |
| 271 | P7 |  | Severe pyrexia, eye mucus, cough or runny nose OR inactive |  |
| 272 | P8 | Vomiting Child | Vomit, taking even small amount of water |  |
| 273 | P8 |  | Developing nausea. |  |
| 274 | P8 |  | Pyrexia (<38℃ ) |  |
| 275 | P8 |  | Less amount of urine OR darker urine |  |
| 276 | P8 |  | Frequent diarrhea |  |
| 277 | P8 |  | Dry skin or lips |  |
| 278 | P9 | Diarrhea Child | Frequent diarrhea |  |
| 279 | P9 |  | Severe abdominal pain |  |
| 280 | P9 |  | Less amount of urine OR darker urineLess amount of urine OR darker urine |  |
| 281 | P10 | Abdominal pain Child | Continuous pain with fluctuation |  |
| 282 | P10 |  | Vomitting OR nausea OR diarrhea |  |
| 283 | P10 |  | Blood in urine |  |
| 284 | P12 | Stools, abnormal color Child | Blood mixed in half of stool |  |
| 285 | P12 |  | Bloody stool AND pyrexia (>38℃ ) |  |
| 286 | P13 | Ear pain Ear discharge Child | Severe pain unresponsive to analgesics OR unable to sleep by pain |  |
| 287 | P13 |  | Bleeding from ear OR blood mixed in ear drainage |  |
| 288 | P13 |  | Foreign body in ear (insect or something) |  |
| 289 | P14 | Headache Child | Unusual condition from the view point　of parent |  |
| 290 | P15 | Tobacco ingestion Child | No specific symptom after 4 hrs to eat tobacco (>2cm) |  |
| 291 | P17 | Appetite loss Child | Less amount of urine OR darker urine |  |
| 292 | P18 | Head injury Child | Able to stop bleeding on the head injury |  |
| 293 | 25 | Stools, abnormal color | Decreased in urination frequency | From Red to Green |
| 294 | 26 | Urination Excessive Frequent | History of DM |  |
| 295 | 26 |  | Severe thirst |  |
| 296 | 26 |  | Intake large amount of water |  |
| 297 | 26 |  | History of pituitary disease |  |
| 298 | 30 | Hearing difficulty | Ear ringing |  |
| 299 | 30 |  | History of under the loud OR noisy sound |  |
| 300 | 31 | Ear ringing | Difficulty of hearing |  |
| 301 | 34 | Neck pain | Palpable nodes under neck skin |  |
| 302 | 34 |  | Pain, only with motion of neck |  |
| 303 | 38 | Hypertension | Nosebleed |  |
| 304 | 40 | Hyperventilation | Abnormally sleepy at day time (Hyperventilation after apnea at sleep) |  |
| 305 | 46 | Mouth and Tooth problem | Pain in the mouth AND pyrexia |  |
| 306 | P2 | Convulsion Child | Unable to be sure whether the event was seizure or not |  |
| 307 | P6 | Shortness of breath Child | Allergic history |  |
| 308 | P12 | Stools, abnormal color Child | Linear blood on the Stool |  |
| 309 | 10 | Dysarthria | Symptoms like common cold (pyrexia, chilling, headache, cough AND runny nose | From Yellow to Green |
| 310 | 14 | Pyrexia | Pain with urination |  |
| 311 | 14 |  | Ear pain |  |
| 312 | 14 |  | Viginal pain OR discharge |  |
| 313 | 19 | Diarrhea | Pyrexia |  |
| 314 | 19 |  | Travel to foreign country within 1 week1 |  |
| 315 | 21 | Heartburn | Unable to improve symptom, even taking stomach medicine |  |
| 316 | 22 | Hematemesis Bloody stools | Pyrexia (>38℃ ) |  |
| 317 | 23 | Urination pain | History of kidney OR parathyroid disease (possibility of ureter stone) |  |
| 318 | 23 |  | Genital discharge of pus |  |
| 319 | 24 |  | Pain wih urination OR residual urine OR Pyrexia (>38℃ ) |  |
| 320 | 25 | Urine color Abnormal | Dark urine AND yellow at skin or white eyes |  |
| 321 | 25 |  | Red urine |  |
| 322 | 25 |  | History of abdominal, pelvic or spine injury OR surgery |  |
| 323 | 26 | Urination Excessive Frequent | Pain with urination OR feeling of residual of urine OR pyrexia |  |
| 324 | 26 |  | History of kidney OR parathyroid disease |  |
| 325 | 26 |  | Back pain OR abdominal pain |  |
| 326 | 27 | Vaginal bleeding | After menopause |  |
| 327 | 27 |  | History of a gynecologic surgery |  |
| 328 | 28 | Genital Urinary problem | Discharge of pus from urethra |  |
| 329 | 28 |  | Rash or lump on penis OR scrotum |  |
| 330 | 28 |  | Itchy on penis OR scrotum |  |
| 331 | 29 | Ear pain Ear discharge | Ear ringing OR difficulty of hearing |  |
| 332 | 30 | Hearing difficulty | Ear fullness |  |
| 333 | 31 | Ear ringing | Continuous ear ringing |  |
| 334 | 34 | Neck pain | Palpable nodes under neck skin |  |
| 335 | 40 | Hyperventilation | Strong feeling of anxiety OR emotional lability |  |
| 336 | 40 |  | History of same episode |  |
| 337 | 45 | Nose injury Nose bleed | Sweling of nose |  |
| 338 | 45 |  | Pain of nose |  |
| 339 | 45 |  | Bruise trace OR scratch OR incision wound, on the nose |  |
| 340 | 45 |  | Clogged nose |  |
| 341 | 46 | Mouth and Tooth problem | General fatigue OR sick OR bad condition |  |
| 342 | 46 |  | White spots OR blisters |  |
| 343 | 46 |  | Severe pain on jaw |  |
| 344 | 46 |  | Pain with biting OR opening of mouth (including jaw pain) |  |
| 345 | 46 |  | Continuous pain, even with usual mouth care |  |
| 346 | 46 |  | History of medication of anticoagulation |  |
| 347 | 46 |  | Toothache AND broken tooth without trauma injury |  |
| 348 | 52 | Bruise wound | Many traces of bruise OR unknown cause of injury |  |
| 349 | 61 | Ear Injury Foreign body | Injury OR swelling of the ear |  |
| 350 | 65 | Foreign body, swallowing of solid mass | Uncomfortable feeling on the throat |  |
| 351 | 72 | Throat Fish bone | Continuous pain and uncomfortable feeling on the throat |  |
| 352 | 79 | Lice | Continuous itchy OR rash at sleep |  |
| 353 | 79 |  | Continuous rash (> 1 week), even with treatment |  |
| 354 | 79 |  | Progressive injury OR suppurative wound |  |
| 355 | 79 |  | Discovery of new eggs after treatment |  |
| 356 | 79 |  | Recurrent new rash after mitigation of rash |  |
| 357 | 79 |  | Allergic reaction aftaer medication of over-the-counter drug OR Prescription drug |  |
| 358 | 79 |  | Pregnant |  |
| 359 | 79 |  | Pyrexia OR nausea OR swelling of lymph node |  |
| 360 | P3 | Cough Child | Cough with sputum |  |
| 361 | P3 |  | Mild hemoptysis with cough |  |
| 362 | P3 |  | Pyrexia (>38℃) |  |
| 363 | P5 | Asthma Child | Pyrexia (>38℃) |  |
| 364 | P5 |  | Yellowish OR greenish sputum |  |
| 365 | P8 | Vomiting Child | Not vomiting now AND still feeling sick |  |
| 366 | P8 | Vomiting Child | Large population with same symptom in family, neighborhood, kindergarten OR school |  |
| 367 | P12 | Stools, abnormal color Child | Whitish stool |  |
| 368 | P13 | Ear pain Ear discharge Child | Discharge of pus from ear hole |  |
| 369 | P17 | Appetite loss Child | Pain in the mouth OR stomatitis |  |
| 370 | P17 |  | Abdominal distension |  |
| 371 | P18 | Head injury Child | Stable after vomit one time |  |
