# Supplementary material for: Revision of the Protocol of the Telephone Triage System in Tokyo, Japan
Source: Emerg Med Int. 2021 Apr 21;2021:8832192. doi: 10.1155/2021/8832192 (PMC8081606; doi:10.1155/2021/8832192)
Supplement: Supplementary Materials — We show three kinds of supplementary files about codes moved to less urgent, codes moved to more urgent, and new codes. [file 8832192.f1.zip › 8832192.f1/Supplement 1 Code moved to more urgent.docx]

| No | ProtcolFileNo | Protocol Name | Code | Change category from A to B |
| --- | --- | --- | --- | --- |
| 1 | 22 | Hematemesis Bloody stools | Did you have a large amount of bright red blood with bowel movement (the largest ever)? | From Orange to Red |
| 2 | 27 | Vaginal bleeding | Do you have a pain on your lower abdomen? |  |
| 3 | 32 | Dizziness, Vertigo | I have a headache. The patient has a headache. Headache. |  |
| 4 | 33 | Numbness | One side of the body is paralyzed. |  |
| 5 | 33 |  | I have dizziness and nausea. OR I have been vomiting. The patient has dizziness and nausea. OR The patient has been vomiting. Dizziness and nausea. OR Vomiting. |  |
| 6 | 37 | Allergy | Have you ever had an anaphylaxis from the same allergen before? |  |
| 7 | 37 |  | Do you have a continous nausea, vomiting, or diarrhea? |  |
| 8 | 37 |  | Any vision changes? |  |
| 9 | 37 |  | Does the patient look confused (agitated or weired response to verbal communication)? |  |
| 10 | 47 | Hand/Wrist problem | I cannot move my arm. |  |
| 11 | 55 | Bites | The patient has skin rash of sudden onset on other part of skin than the animal/insect bite. |  |
| 12 | 55 |  | The patient has severe pain and swelling on the skin surrounding the site of animal/insect bite. |  |
| 13 | 55 |  | The patient has urticaria/hives of sudden onset. |  |
| 14 | 55 |  | There is sudden swelling on the other limbs than animal/insect bite. |  |
| 15 | 55 |  | I have difficulty moving due to the severe pain. |  |
| 16 | 55 |  | I have sudden rash, itchiness, swelling on other sites than the animal/insect bites. |  |
| 17 | 55 |  | The patient has muscle cramps, abdominal pain, or extreme anxiety (different from normal state). |  |
| 18 | 55 |  | The patient has nausea/vomiting or abdominal cramps. |  |
| 19 | 55 |  | Have you ever had an allergic reaction to ticks? |  |
| 20 | 56 | Burns | The burn goes round the full circumference of the neck, arm, or leg (except for sunburn). |  |
| 21 | 63 | Trunk injury | Is the chest/abdominal pain worsening? |  |
| 22 | P11 | Constipation Child | There is a large amount of blood component in stool? OR The stool contains strawberry jam like components? |  |
| 23 | 37 | Allergy | Do you have a swelling of your face or limbs? | From Yellow to Red |
| 24 | 15 | Rash | Do you have a rapidly progressive rashes or itchness? | From Yellow to Orange |
| 25 | 36 | Itch | Do you have a rash or itching that intensifies rapidly? |  |
| 26 | 52 | Bruise wound | Do you have severe pain in your hands or feet? |  |
| 27 | 53 | Falls | Do you feel dizzy or nauseous? |  |
| 28 | 55 | Bites | Is the needle still remaining on the skin? |  |
| 29 | 55 |  | Do you have a fever, chill, or sweating? |  |
| 30 | 62 | Neck and Back injury | Has it been more than 12 hours since the injury and have you started to feel dizzy or nauseous? |  |
| 31 | 63 | Trunk injury | More than 12 hours have passed since the time of injury AND the patient has a gradual onset of dizziness or nausea. |  |
| 32 | 5 | Disturbance of consciousness | The patient seems to be in usual condition, showing normal response. Apparently in usual condition with normal response. | From Green to Yellow |
| 33 | 9 | Back pain | I have a fever. The patient has a fever. Fever. |  |
| 34 | 37 | Allergy | Is your nausea/vomiting/diarrhea tolerable? |  |
| 35 | 37 |  | Do you have a mild rash or itchness? |  |
