# Supplementary material for: Revision of the Protocol of the Telephone Triage System in Tokyo, Japan
Source: Emerg Med Int. 2021 Apr 21;2021:8832192. doi: 10.1155/2021/8832192 (PMC8081606; doi:10.1155/2021/8832192)
Supplement: Supplementary Materials — We show three kinds of supplementary files about codes moved to less urgent, codes moved to more urgent, and new codes. [file 8832192.f1.zip › 8832192.f1/Supplement 1 New Code.docx]

| No | ProtcolFileNo | Protocol Name | Code | New category |
| --- | --- | --- | --- | --- |
| 1 | 1 | Shortness of breath | The patient has a facial swelling, redness or hives. | Red |
| 2 | 4 | Palpitations | The patient has a racing heart, or a slow heartbeat. |  |
| 3 | 8 | Chest pain | Did your pain start all of a sudden? |  |
| 4 | 12 | Syncope | The patient has a headache, a chest pain, a back pain, or a difficulty in breathing? |  |
| 5 | 14 | Pyrexia | The patient has a headache and a lethargy, or an altered mental status. |  |
| 6 | 15 | Rash | The patient has a pain and a swelling. |  |
| 7 | 18 | Constipation | Do you have nausea/vomiting with fever > 38C ? |  |
| 8 | 19 | Diarrhea | The patient feels severe generalized weakness. |  |
| 9 | 20 | Vomiting | The patient has a bad headache. |  |
| 10 | 21 | Heartburn | The patient has nausea or palpitation, with 65 years or older, diabetes, AMI, angina, post CABG, cerebrovascular accident, or taking aspirin. |  |
| 11 | 27 | Vaginal bleeding | Are you currently >/= 12 weeks pregnant? |  |
| 12 | 32 | Dizziness, Vertigo | Did your dizziness start all of a sudden? |  |
| 13 | 33 | Numbness | Close your eyes and extend both of your arms forward, horizontally to the floor, with your palms upwards. Do one or both of your arms drop or rotate internally? |  |
| 14 | 33 |  | The patient has has facial asymmetry, or difficulty moving/drooping one side of his/her face. |  |
| 15 | 33 |  | The numbness started all of a sudden. |  |
| 16 | 34 | Neck pain | Did your pain reach its maximal strength all at once? |  |
| 17 | 41 | Anxiety | Suicide attempt (self-damage / drug addiction). Fall / stab, dangerous drug poisoning, shock, etc. |  |
| 18 | 42 | Insomnia | Suicide attempt (self-damage / drug addiction). Fall / stab, dangerous drug poisoning, shock, etc. |  |
| 19 | 43 | Depression | Suicide attempt (self-damage / drug addiction). Fall / stab, dangerous drug poisoning, shock, etc. |  |
| 20 | 47 | Hand/Wrist problem | The patient's arm is deformed or direction of my arm is abnormal. |  |
| 21 | 51 | Hand/Wrist problem | The patient is bleeding heavily and it is difficult to stop bleeding. |  |
| 22 | 73 | Foreign body, Rectum | There is strong pain other than the anus, such as the patient's abdomen and shoulders (radiating pain: non-local pain). |  |
| 23 | 78 | Hypothermia | Patient does not move or loses consciousness. |  |
| 24 | 80 | Motionless | The patient's symptoms are progressing rapidly |  |
| 25 | P3 | Cough Child | The patient cannot lie down, walk, or speak due to cough. |  |
| 26 | P5 | Asthma Child | Did the patient cough out a large amount of blood? |  |
| 27 | P6 | Shortness of breath Child | The patient cannot lie down, walk, or speak? |  |
| 28 | P10 | Abdominal pain Child | The patient is exhausted and weak? |  |
| 29 | P10 |  | The patient is lethargic? OR The patient is saying unusual things? OR The patient is crying and agitated? (The patient cannot answer normally to verbal communication) |  |
| 30 | P12 | Stools, abnormal color Child | The patient is exhausted and weak? |  |
| 31 | P14 | Headache Child | The patient is lethargic. OR The patient says abnormal things. (The patient cannot respond appropriately to verbal communication) |  |
| 32 | P18 | Head injury Child | My kid had seizure. |  |
| 33 | P18 |  | My kid is lethargic or acting abnormally (ex. Cannot respond normally on verbal communication). |  |
| 34 | P18 |  | He/she has a large amount of bleeding from his/her head, ear(s), nose (Bleeding dose not stop with compression)? |  |
| 35 | P18 |  | He/she is agitated or exhausted (he/she still can be woken up). |  |
| 36 | 33 | Numbness | The patient has an irregular gasp, dizziness, or trembling/numbness in the hands/face/lips. | Orange |
| 37 | 41 | Anxiety | Suicide attempt (self-damage / drug addiction). Wrist cuts that require hemostasis, drug overdose, etc. |  |
| 38 | 42 | Insomnia | Suicide attempt (self-damage / drug addiction). Wrist cuts that require hemostasis, drug overdose, etc. |  |
| 39 | 43 | Depression | Suicide attempt (self-damage / drug addiction). Wrist cuts that require hemostasis, drug overdose, etc. |  |
| 40 | 47 | Hand/Wrist problem | The patient does not move the injured arm. He/she has pain when trying to move injured arm. |  |
| 41 | 51 | Laceration | The patient was injured by a contaminated material. |  |
| 42 | P1 | Pyrexia Child | My baby looks sleepy for longer than 8 hours (he/she can still respond when addressed). |  |
| 43 | P1 | Pyrexia Child | My baby looks exhausted. |  |
| 44 | P2 | Convulsion Child | The patient is irritable even after he/she gained consciousness? |  |
| 45 | P3 | Cough Child | The patient's breath is faster than usual. |  |
| 46 | P5 | Asthma Child | Does the patient have difficulty sleeping due to shortness of breath? |  |
| 47 | P5 |  | Did the symptom start all of a sudden? |  |
| 48 | P5 |  | Did the symptom start after eating something, taking a medication, or getting insect bite? |  |
| 49 | P6 | Shortness of breath Child | The patient cannot sleep due to cough? |  |
| 50 | P6 |  | The symptom starterd all of a sudden? |  |
| 51 | P6 |  | The symptom started after eating, taking medications, or insect bite? |  |
| 52 | P6 |  | The patient's breath looks shallower and faster than usual? |  |
| 53 | P6 |  | The patient cannot take water orally? The patient has nausea/vomiting? |  |
| 54 | P8 | Vomiting Child | My Kid/The patient has not peed/urinated for more than half a day. |  |
| 55 | P9 | Diarrhea child | The patient is vomiting MANY times (MANY means: more than 5 times OR more than twice when you no longer see residual food in the vomitus)? |  |
| 56 | P9 |  | The patient looks sleepy for more than 8 hours (patient can answer to verbal communication)? |  |
| 57 | P9 |  | NO urine is seen for 8-12 hours. (sign of dehydration) |  |
| 58 | P10 | Abdominal pain Child | The abdomen is very painful even with a slight pressure? |  |
| 59 | P10 |  | The patient is vomiting MANY times (MANY means: more than 5 times OR more than twice when you no longer see residual food in the vomitus)? |  |
| 60 | P10 |  | The patient looks sleepy for more than 8 hours (patient can answer to verbal communication)? |  |
| 61 | P10 |  | NO urine is seen for 8-12 hours. (sign of dehydration) |  |
| 62 | P11 | Constipation Child | Patient's abdomen is severely painful when touched? |  |
| 63 | P12 | Stools, abnormal color Child | The patient is vomiting MANY times (MANY means: more than 5 times OR more than twice when you no longer see residual food in the vomitus)? |  |
| 64 | P14 | Headache Child | The patient vomited more than twice. |  |
| 65 | P14 |  | The patient looks sleepy for more than 8 hours (still able to respond when addressed). |  |
| 66 | P16 | Crying Child | The patient is suffering so severely that he/she cannot walk. |  |
| 67 | P18 | Head injury Child | My Kid/The patient vomited several times. Vomiting several times. |  |
| 68 | P18 |  | There was the loss of consciousness after/with injury. |  |
| 69 | P18 |  | Does the swelling feel soft/flaccid when you touch it? |  |
| 70 | P18 |  | The mechanism of injury was high risk (fall from > 1m height, hit by a motor vehicle, etc). |  |
| 71 | 14 | Pyrexia | The patient has been with a fever for more than 2 days. | Yellow |
| 72 | 21 | Heartburn | The patient has nausea or palpitations. |  |
| 73 | 24 | Difficulty Urination | Do you have any signs of dehydration? |  |
| 74 | 24 |  | Do you have a pain of distention of the lower abdomen? |  |
| 75 | 27 | Vaginal bleeding | Are you currently < 12 weeks pregnant? |  |
| 76 | 41 | Anxiety | Suicide attempt (self-damage / drug addiction). Wrist cuts that do not require hemostasis, drug overdose, etc. |  |
| 77 | 42 | Insomnia | Suicide attempt (self-damage / drug addiction). Wrist cuts that do not require hemostasis, drug overdose, etc. |  |
| 78 | 43 | Depression | Suicide attempt (self-damage / drug addiction). Wrist cuts that do not require hemostasis, drug overdose, etc. |  |
| 79 | 55 | Bites | I have a fever. |  |
| 80 | 55 |  | Do you have redness, pain, swelling, or discharge of liquid from the site of animal bite? |  |
| 81 | 55 |  | I have diabetes. |  |
| 82 | 57 | Wound infection | The patient has just been injured |  |
| 83 | 62 | Neck and Back injury | The patient has wounds. |  |
| 84 | 73 | Foreign body, Rectum | The patient has a swollen area |  |
| 85 | 80 | Motionless | The patient has a reduced appetite. |  |
| 86 | 80 |  | The patient is becoming unable to walk. |  |
| 87 | P1 | Pyrexia Child | No tears are seen in his/her eyes. His/her mouth/tongue looks dry. |  |
| 88 | P1 |  | My kid feel pain when urinating. I see any abnormalities in his/her urinary odor/color. |  |
| 89 | P1 |  | My kid recently (<1month) travel abroad (except for north America or Europe). |  |
| 90 | P2 | Convulsion Child | The patient cannot take a sufficient amount of water orally? (The amount of water intake is decreasing) |  |
| 91 | P2 | Convulsion Child | There is a decrease in the amount of urine or dark color change of urine? (dehydration) |  |
| 92 | P2 | Convulsion Child | The patient had a seizure without a fever? |  |
| 93 | P3 | Cough Child | The patient cannot take a sufficient amount of water orally? (The amount of water intake is decreasing) |  |
| 94 | P3 |  | My kid recently (<1month) travel abroad (except for north America or Europe). |  |
| 95 | P4 | Runny nose Child | The patient is older than 1 year and seems suffered from stuffy nose. |  |
| 96 | P4 |  | The patient cannot take water sufficiently. |  |
| 97 | P4 |  | The patient seems dizzy or imbalanced. |  |
| 98 | P4 |  | There are a redness and a swelling of the cheak and a fever. |  |
| 99 | P4 |  | The symptoms do not go away. |  |
| 100 | P5 | Asthma Child | Does the patient look different from his/her usual state? |  |
| 101 | P7 | Rash Child | The symptoms started after eating, taking medication(s), or insect bite? |  |
| 102 | P7 |  | My kid recently (<1month) travel abroad (except for north America or Europe). |  |
| 103 | P8 | Vomiting Child | My kid recently (<1month) travel abroad (except for north America or Europe). |  |
| 104 | P9 | Diarrhea child | The patient is vomiting? |  |
| 105 | P9 |  | People around the patient (femily, neighborhood, nursery school, kindergarten, school etc) have the same symptoms? |  |
| 106 | P9 |  | People who had the same meal complain the same symptoms? |  |
| 107 | P9 |  | The patient recently ate outside, raw foods, or any food which had not been preserved well? |  |
| 108 | P9 |  | The patient had vomiting more than 2 times? |  |
| 109 | P9 |  | The patient recently (<1 month) traveled abroad (except for North America or Europe)? |  |
| 110 | P10 | Abdominal pain Child | The patient cannot take a sufficient amount of water? |  |
| 111 | P10 |  | People who had the same meal complain the same symptoms? |  |
| 112 | P10 |  | The patient recently ate outside, raw foods, or any food which had not been preserved well? |  |
| 113 | P10 |  | The patient hit his/her abdomen? There are any wounds on the abdomen? |  |
| 114 | P11 | Constipation Child | The patient cannot take a sufficient amount of water? OR The patient vomits after taking water? |  |
| 115 | P11 |  | The patient cannot sleep due to the symptoms. |  |
| 116 | P12 | Stools, abnormal color Child | The patient has nausea/vomiting. |  |
| 117 | P12 |  | The patient traveled abroad less than 1 month ago. |  |
| 118 | P14 | Headache Child | The patient looks different from usual based on observation by his/her guardian(s) |  |
| 119 | P15 | Tobacco ingestion Child | The patient looks different from usual based on observation by his/her guardian(s) |  |
| 120 | P16 | Crying Child | The patient cannot take water sufficiently. |  |
| 121 | P16 |  | Do you see any potential sources of pain on the whole body of the patient? For example, a hair or fiber tangled around a finger etc). |  |
| 122 | P18 | Head injury Child | Does he/she look different from his/her usual condition? |  |
| 123 | P18 |  | Do you see any swelling/lump on anywhere other than forehead? |  |
| 124 | 40 | Hyperventilation | Do you have a generalized weakness? | Green |
| 125 | 44 | Eye problems | The patient has only red white eyes and no other symptoms |  |
| 126 | 73 | Foreign body, Rectum | Bleeding has stopped and the pain is mild |  |
| 127 | 80 | Motionless | Not applicable to the above symptoms, but the patient feels anxious |  |
| 128 | P12 | Stools, abnormal color Child | The patient has a diarrhea. |  |
